# Book review by Dr Sebaka Malope

**DOI:** 10.4102/phcfm.v12i1.2410

**Published:** 2020-06-17

**Authors:** Sebaka Malope

**Affiliations:** 1 Department of Family Medicine, Family Medicine Specialty Training Programme, Lesotho Boston Health Alliance, Maseru, Lesotho

This multi-authored book is the real stimulus for interest in primary care and research. It starts with compelling discussions highlighting the merits and gaps to be addressed only via primary care research. It convinces one of the importance of contextually appropriate evidence for primary care. It further highlights epistemological position that may be unique for this setting, which may allow different methodologies that could have otherwise been applied individually in a different setting to be used to enrich findings.

The thoughtful weaving of the storyline in the film *The Matrix* cleverly sheds light on different lenses (ontological and epistemological positions) that researchers can take whilst tackling primary care issues through research. The story also compels one to think harder not to disregard these positions before embarking on primary care research. This is the clever way of capturing graphically the readers into these concepts and I find them difficult to forget.

The book also dispels wrong assumptions and puts into perspective terminologies that are otherwise used loosely in research. The clear example of this is in chapters 4 and 5 where interdisciplinary approach is differentiated from mixed-methods approach. The examples used in these chapters are compelling and successfully drive the idea of interdisciplinary research home.

The book is reader-friendly and practical whilst ensuring important themes of research in primary care are thoroughly explained. Multiple author nature allows the readers to see primary care research from different primary care contextual positions, even so through examples provided to discuss each subject. Although research terminology is used throughout the book, the key is application in the complexity of primary care setting.

Another important subject described in this book is quality improvement in primary care, how it contrasts with traditional research and important gaps to be addressed through development of this field. This is an important area for African primary care practitioners whose systems are bugged with challenges requiring leadership in the area of clinical governance.

It is also the strength of this book that the chapters are precise and not lengthy to guide individuals through important steps in primary care research. The resource boxes in each chapter are also very useful for individuals who want to learn deeper into certain aspects of their research that needs further reading.

I find this book useful, practical and user-friendly. I recommend it as a resource for individuals who are starting their journey in research, especially young primary care researchers, residents and teachers of family medicine.

